# Adding sildenafil vaginal gel to clomiphene citrate in infertile women with prior clomiphene citrate failure due to thin endometrium: a prospective self-controlled clinical trial

**Published:** 2017-03

**Authors:** AN Fetih, DM Habib, II Abdelaal, M Hussein, GN Fetih, ER Othman

**Affiliations:** Department of Obstetrics and Gynecology, Faculty of Medicine, Assiut University, Egypt; Department of Pharmaceutics, Faculty of Pharmacy, Assiut University, Egypt

**Keywords:** Anovulation, clomiphene citrate, endometrium, endometrial thickness, infertility, sildenafil vaginal gel

## Abstract

We aimed to investigate the effect of adding sildenafil vaginal gel to clomiphene citrate (CC) in infertile women with prior CC failure.

**Methods:**

This is a self- controlled clinical trial. Women with CC failure (in prior 5 cycles) and thin endometrium were recruited (N = 42). In their 6th (CC only) cycle, women continued on CC 100 mg/ day for 5 days, and had measurement of endometrial thickness and Doppler assessment of uterine arteries on day of HCG administration. In the 7th cycle, women (N = 36) were given usual dose of CC supplemented with sildenafil vaginal gel (5 gm, containing 50 mg sildenafil) twice daily from cycle day 8 to day of HCG administration. Endometrial thickness and uterine artery Doppler were measured on the day of HCG injection.

**Results:**

In the 7th (CC + sildenafil vaginal gel) cycle, endometrial thickness was significantly higher than in the 6th (CC only) cycle (9.3 mm +/- 3.1mm versus 6.6 mm +/- 1.4 mm, respectively, P = < 0.001). Uterine artery pulsatility index dropped from 2.4 +/- 0.8 in 6th cycle to 1.6 +/- 1.3 in 7th cycle (P = 0.002). Clinical pregnancy rate increased but numbers were too small (only 3 pregnancies).

**Conclusion:**

Sildenafil vaginal gel significantly increased endometrial thickness and uterine blood flow, and may improve pregnancy rate in patients with CC failure due to thin endometrium. Mucoadhesive vaginal gel formulation allowed shorter duration of sildenafil application, and less frequent daily dosing.

## Introduction

Anovulation is a cause of infertility in 20% of the cases (Weiss et al., 2014). Around 85% of the women with anovulatory infertility have normal gonadotropin levels and are classified as type-2 anovulation according to the world Health Organization classification (The ESHRE Capri Workshop Group, 2012). Most of these cases are the result from polycystic ovarian syndrome (PCOS) (Brown et al., 2009).

Guidelines state that clomiphene citrate (CC) is the first line treatment for ovulation induction in women with type 2 anovulation or polycystic ovaries (The Thessaloniki ESHRE/ASRM-Sponsored PCOS Consensus Workshop Group, 2008; Brown et al., 2009; The ESHRE Capri Workshop Group, 2012). CC is a non-steroidal compound that works as anti-oestrogen. It blocks oestrogen receptors at the hypothalamus, releasing it from negative feedback, and augmenting its release of GnRH. Subsequently, pituitary production of gonadotropins is increased resulting in follicular growth, and ovulation (Balen, 2013). Ovulation rates with CC approach 70- 85% per cycle, whereas cumulative pregnancy rates in a 6-month period is 40- 70% (Eijkemans et al., 2003; Van Santbrink and Fauser, 2006).

About 15% of anovulatory women do not respond to CC (Brown et al., 2009). This is identified as clomiphene citrate resistance. On the other hand, clomiphene citrate failure refers to another subset of women who do not get pregnant despite achieving ovulation after CC treatment (The Thessaloniki ESHRE/ASRM-Sponsored PCOS Consensus Workshop Group, 2008; Balen, 2013). Management of women with clomiphene citrate resistance or failure consists of gonadotropin induction of ovulation or laparoscopic ovarian drilling in PCOS (Van Santbrink and Fauser, 2006). Ovulation induction with gonadotropins is expensive, requires frequent monitoring, is associated with higher risk of ovarian hyperstimulation syndrome, and multiple pregnancies (Eijkemans et al., 2003) Similarly, laparoscopic ovarian drilling is associated with potential damage to ovarian reserve, and possible adhesion formation (Fernandez et al., 2011). Therefore, analysis of factors leading to clomiphene citrate failure is necessary to make full use of this inexpensive, relatively safe, and effective method of ovulation induction.

An important cause of clomiphene citrate failure is its anti-estrogenic effect on the endometrium (Dehbashi et al., 2003; Homburg et al., 2012). As oestrogen is necessary for endometrial growth during the follicular phase of the cycle, the antiestrogenic effects of CC impair endometrial growth (Amita et al., 2010). This is evident in endometrial biopsies from women on CC, which show impaired epithelial proliferation, decreased number and diameter of glands, with delayed glandular maturation (Bonhoff et al., 1996; Sereepapong et al., 2000). Sonographically, this poor endometrial development is identified as thin endometrium (Nakanrura et al., 1997).

Oestrogen mediated growth of the endometrium is dependent on the blood flow to the endometrium (Sher and Fisch, 2002). It is then logical to think of vasodilator therapies as potential solutions to improve endometrial blood flow and thickness in infertile women with clomiphene citrate failure.

Sildenafil is a type 5-phosphodiestrase inhibitor that augments vasodilator effect of nitric oxide on vascular smooth muscles by preventing the degradation of cGMP (Sher and Fisch, 2000, 2002). Sildenafil citrate could lead to an improvement in uterine blood flow and, in conjunction with oestrogen, it leads to the oestrogen-induced proliferation of the endometrium (Richter et al., 2007).

Few studies addressed the use of sildenafil to increase endometrial thickness in CC stimulated cycles (Malinova et al., 2013; Das et al., 2009). In these studies, sildenafil was given as vaginal suppositories and in multiple doses per day. In addition, it was not clear whether the studied population had persistently thin endometrium with prior cycles of CC induction.

In the present study, we evaluate the use of a new formulation of topical sildenafil (sildenafil vaginal gel) to increase endometrial thickness and uterine blood flow in women with clomiphene citrate failure due to thin endometrium.

## Materials and Methods

### Study design

This is a prospective self-controlled clinical trial. Clinicaltrial.gov identifier is NCT02710981. Assiut University Institutional Review Board approved the study (IRB approval # 00009898)

### Study subjects

We counselled and recruited 42 infertile women who had been diagnosed with anovulatory infertility, with normal baseline FSH, LH, and free testosterone levels, patent tubes on hysterosalpingography, and normal male semen analysis.

By the time they were enrolled in the study, all women had already completed 5 ovulatory cycles of CC induction (in a dose of 100 mg/ day in two divided doses starting from day 3 of the cycle for 5 days) but without conception (clomiphene citrate failure) with thin endometrium (<8mm) in at least 3 cycles (Takasaki et al., 2010). Ovulation was documented sonographically and with day-21 serum progesterone exceeding 5-ng/ ml in all cases. Women who agreed to be enrolled in the study signed an informed consent. All enrolled cases were ovulation induction, not IUI cycles.

All procedures performed in our study were in accordance with the ethical standards of the institutional and/or national research committee (IRB # 00009898), and with the 1964 Helsinki declaration and its later amendments (Tokyo, 2004). Informed consent was obtained from all individual participants included in the study.

### Exclusion criteria

Women with major medical problems, male factor infertility, endocrine abnormalities such as hyperprolactinemia or abnormal thyroid functions, prior ovarian or adnexal surgery, or organic pelvic pathology (fibroids, adenomyosis, or congenital uterine anomalies) were not included in the study.

### Intervention

Sixth cycle (CC only cycle)

Enrolled cases were asked to continue with CC induction (sanofi-aventis, NJ, USA) at the same dose, for one more cycle (the 6th cycle). When the leading follicle reached 18 mm in diameter, 10000 IU of highly purified HCG (IBSA, Lugano, Switzerland) was prescribed for IM injection. Within the 6th cycle, a gel base, without medicament (vehicle only), was applied vaginally in a dose of 5 gm twice per day, from cycle day 8 to the day of HCG injection. Transvaginal sonography was done to assess endometrial thickness and uterine artery Doppler indices on the day of HCG injection.

Seventh cycle (CC + Sildenafil vaginal gel cycle)

Those who didn’t conceive on the sixth cycle (41 women) were asked to have an extra (7th) cycle in which they were prescribed the usual dose of CC, in addition to sildenafil vaginal gel. The sildenafil vaginal gel consists of sildenafil acetate as a medicament (Sigma Aldrich, St. Louis, MO, USA) loaded on an in situ gelling system based on two polymers: Pluronic p-127 (BASF Ltd., Ludwigshafen, Germany), and Hydroxy Ethyl Cellulose (Sigma Aldrich, St. Louis, MO, USA). The dose was 5 g gel containing 50 mg sildenafil, applied vaginally twice daily from cycle day 8 to the day of HCG administration. The gel was prepared in collaboration with the department of pharmaceutics, faculty of Pharmacy, Assiut University. Sixty grams of gel were prepared in tubes and every patient was given 12 applicators like that used for administration of vaginal antimycotic creams to use them for applying the gel. Only 36 women agreed to go for the CC + sildenafil vaginal gel cycle, whereas 5 women withdrew consent as they preferred to go for other forms of fertility treatment (gonadotropin induction or IVF/ICSI). No estradiol or any other adjuvants were added in either the 6th or 7th treatment cycles apart from those specified above.

### Evaluation of the study end points

The ultrasonography machine used in this study is Medison, SonoAce X8. A transvaginal probe with a frequency of 4 to 9 MHz (Medison 3D4-9ES) was used to examine the cases (Samsung-Medison, Seoul, South Korea). Only two experienced operators (MHM, and DMH) evaluated all cases in their 6th and 7th cycles according to the study operational manual.

During the 6th and 7th cycle, endometrial thickness and uterine artery Doppler assessment were done on the day of HCG administration. Endometrial thickness measurement was done through measuring the maximal distance spanning endometrialmyometrial interphase on each endometrial stripe in a sagittal plane at the region of the uterine fundus. Transvaginal Doppler examination of the pulsatility indices of both uterine arteries was also done during the same visit. Uterine arteries were visualized by colour Doppler lateral to the cervix with the angle of insonation of 30˚. Pulsatility index (PI), as a measure of resistance to blood flow in uterine arteries, was determined. The mean of PI in both uterine arteries was calculated and recorded.

Although pregnancy rate was not a primary outcome measure in our study, we detected pregnancy in 3 cases in our cohort of 36 cases of infertile women with clomiphene failure in which sildenafil gel was added as an adjuvant. Pregnancy was diagnosed with a β-HCG value above 100 mIU/ ml, and confirmed later with ultrasound at 7-8 weeks of gestation.

## Statistics

Data were analysed using SPSS program ver. 17. Clinical and demographic characteristics of the enrolled cases were expressed as mean ± standard deviation. Student-t-test was used to compare continuous variables, while Fisher’s exact test compared categorical data in the 6th and 7th cycle.

## Results

We recruited 42 cases of infertile women, with clomiphene citrate failure (6 ovulatory cycles, with no conception), and thin endometrium (in at least 3 of the CC stimulated cycles). Thirty six patients attempted a seventh cycle with sildenafil vaginal gel as an adjuvant treatment prescribed on cycle day 8 to the day of HCG administration, added to the usual dose of CC used for ovulation induction. Clinical and basic laboratory characteristics of our patient cohort are outlined in Table I.

**Table I:** — Clinical and basic laboratory characteristics of recruited cases

Characteristic	**Value**
Age	26.8 ± 2.8 years , (range =22-33 years)
BMI	29.1 ± 4.6 kg/m2
Serum FSH	4.5 ± 1.5 U/ L, (patient range = 2.1-7.5)
Serum LH	9.9 ± 4.8 U/ L, (patient range =2.0 – 20.0)
Serum free testosterone	0.77 ± 0.23 ng/ dl, (patient range = 0.4-1.2 ng/ml).
Primary infertility	N = 26, (72.22 %)
Secondary infertility	N = 10, (27.77%)
Duration of infertility	4.3 ±1.2 years Range: 2 – 7 years
Mean parity	0.3 ± 0.1

Follicular number and size (measured on the day of HCG injection), and number of cycle days until HCG injection were comparable for our cohort of women in their CC only cycle (6th cycle), and the CC + sildenafil vaginal gel cycle (7th cycle). Mean follicular number was 1.3 in the sixth cycle, and 1.4 in the seventh cycle (P value = 0.38). Mean follicular diameter was 17.8 versus 18.8 during the sixth and seventh cycle respectively (P value = 0.096), as seen in Table II.

**Table II:** — Cycle characteristics and pregnancy outcome in the sixth (CC alone) and the seventh (CC + sildenafil vaginal gel) cycles of treatment (N/A: Not applicable due to very few numbers of pregnancies)

**Cycle characteristics / outcome**	**Sixth cycle (CC only cycle)**	**Seventh cycle (CC + sildenafil vaginal gel cycle)**	**P value**
Number of follicles	1.3 +/- 0.5	1.4 +/- 0.6	0.38
Follicular diameter	17.8 +/- 1.3	18.8 +/- 1.7	0.096
Number of cycle days till HCG injection	12.0 ± 0.2	11.8 ± 0.18	0.7
Endometrial thickness	6.6 +/- 1.4 mm	9.3 +/- 3.1mm	< 0.001
Uterine artery Pulsatilty index	2.4 +/- 0.8	1.6 +/- 1.3	0.002
Clinical pregnancy rate per cycle	1 (2.3 %)	3 (8.4%)	N/A

**Figure 1:**
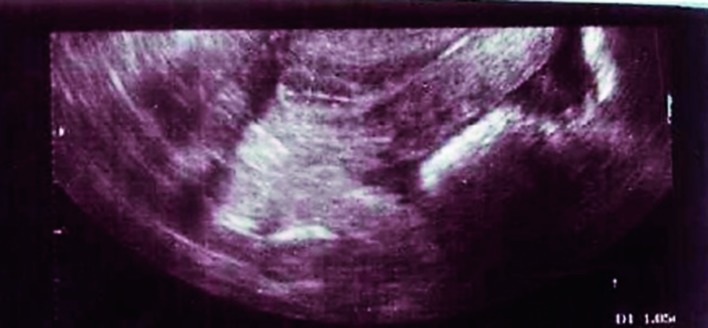
— Endometrial thickness on day of HCG injection in a woman on clomiphene citrate plus sildenafil vaginal gel (7th cycle). Note the triple line endometrium

**Figure 2:**
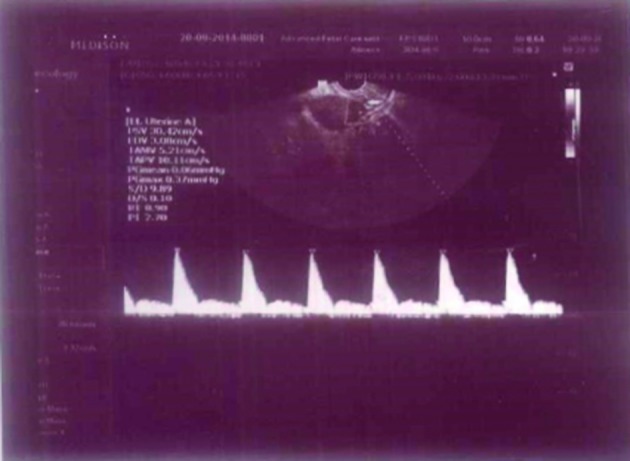
— Doppler waveform on day of hCG injection in a woman under clomiphene citrate treatment (6th cycle)

**Figure 3:**
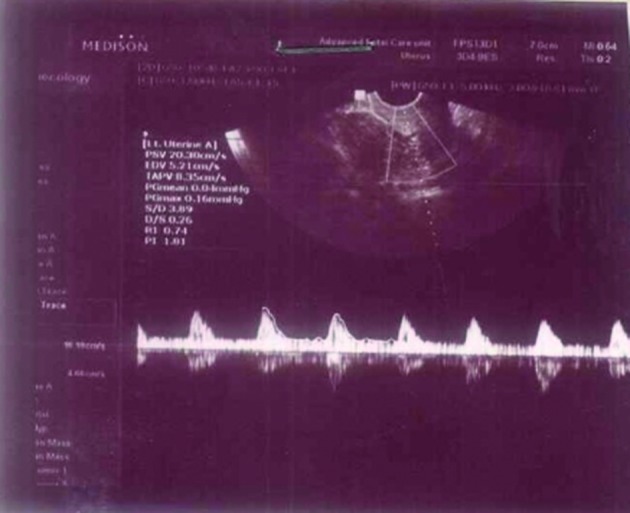
— Doppler waveform on day of hCG injection in a woman under clomiphene citrate plus sildenafil vaginal gel treatment (7th cycle).

Endometrial thickness showed a statistically significant increase from a value of 6.6 +/- 1.4 mm with CC only treatment during the sixth cycle, to 9.3+/- 3.1mm with CC+ sildenafil vaginal gel in the seventh cycle. P value was <0.001 (Table II, Fig 1). As an indicator of uterine blood flow, we used Doppler ultrasound to measure the pulsatility index in both uterine arteries. With the addition of the sildenafil vaginal gel, the uterine artery pulsatility index dropped from 2.4 +/- 0.8 to 1.6 +/- 1.3 (P value = 0.002) indicating a significant reduction in blood flow resistance in the uterine arteries (Table II, Fig 2 & 3).

## Discussion

Using CC in ovulation induction is associated with discrepancy between high ovulation rates (70- 80 %), and low pregnancy rate (only 10- 20 % per cycle) (Badawy et al., 2007; Badawy et al., 2008(. This is attributed, at least partly, to poor endometrial growth in CC induction cycles because of its antiestrogenic effect at the endometrium (Mitwally and Casper, 2006). Various strategies have been attempted to minimize estrogenic antagonist actions of CC but with limited success (Reynolds et al., 2010).

In the present study, we tested, for the first time to the best of our knowledge, the effects of sildenafil vaginal gel on endometrial thickness in women with CC failure due to thin endometrium. Our results have shown that sildenafil vaginal gel when added to CC increased endometrial thickness significantly, and improved uterine blood flow. We have also shown an increase in pregnancy rate, although the numbers were too small to draw statistical significance.

We used a prospective self-controlled clinical trial design for our study. This will eliminate the confounding effects of factors such as age, BMI, race, socioeconomic class, and genetic background, as each case controls for itself (Grady et al., 2007). In addition, we tested a novel formulation of local sildenafil therapy, which is sildenafil vaginal gel to achieve the desired vasodilator effect at the level of the endometrium, while avoiding systemic side effects.

Lack of a control group is a limiting factor in our study. However, our recruited subjects are highly selected in terms of having CC failure (6 ovulatory cycles without conception), with thin endometrium in at least 3 cycles). Having thin endometrium in more than one cycle before the intervention emphasizes that this is a persistent trend in this group of patients, and minimizes the likelihood of having a “regression to the mean” effect in the subsequent intervention cycle.

In agreement with our findings are previous reports that had shown that addition of sildenafil vaginal suppositories to CC induction of ovulation achieved significantly higher endometrial thickness, reduction in uterine artery vascular resistance, and increase in pregnancy rate (Das et al., 2009; Malinova et al., 2013). In addition, for women with prior failed IVF/ICSI cycles associated with thin endometrium, using sildenafil vaginal suppositories during their subsequent cycles expanded their endometrial growth, enhanced endometrial blood flow, and improved their pregnancy rates (Sher and Fisch, 2000; Sher and Fisch 2002; Richter et al., 2007; Takasaki et al., 2010).

Few studies addressed the relation between endometrial thickness and pregnancy outcome in CC induction cycles. Thin endometrium was associated with non-conception according to some studies (Esmailzadeh and Faramarzi, 2007; Shahin, 2008), while in others preovulatory endometrial thickness was of limited predictive ability for pregnancy (Kolibianakis et al., 2004; Asante et al., 2013). The reason for these contradicting results may be because of different stimulation protocols (CC alone, CC+ HMG, or IVF/ICSI) (Reynolds et al., 2010), different cut-off values to define thin endometrium (6, 7, or 8 mm) (Senturk and Erel, 2008), and variable timing of endometrial thickness measurement (day of HCG administration, or fixed cycle day 10 or 12 (Asante et al., 2013).

Despite this debate, endometrial thickness below 7 mm is generally considered suboptimal for pregnancy (Casper, 2011; Gleicher et al., 2013). Our study findings confirm the importance of endometrial thickness to achieve pregnancy in CC induction cycles, as CC failure was associated with thin endometrium, whereas the increase in endometrial thickness with sildenafil vaginal gel was associated with an increase in clinical pregnancy rate.

The mechanism by which clomiphene citrate causes thinning of the endometrium is unknown. Working as an antagonist at the endometrial oestrogen receptor level is a major cause (Mitwally and Casper, 2006). In addition, diminished uterine and endometrial blood flow in clomiphene citrate stimulated cycles might also play a pathogenic role (Rogers et al., 1998; Ng EH et al., 2007). Sakhavar and colleagues could not find significant difference in uterine artery pulsatility or resistive indices among unexplained infertility patients treated with clomiphene citrate, lotrezole, or control women (Sakhavar et al., 2014). This is taken as a basis for an argument that diminished endometrial blood flow is not a cause for impaired endometrial growth in clomiphene citrate treatment. However, the authors recruited normo-ovulatory women with unexplained infertility. In addition, there was no assessment of endometrial thickness in this study. Consequently, we don’t believe that Sakhavar’s findings could be extrapolated to anovulatory patients with thin endometrium in repeated clomiphene citrate cycles like the population we recruited to our study.

On the other hand, Palomba et al. (2006) reported that PCOS women who ovulated under clomiphene citrate treatment had significantly higher uterine artery resistive index (RI), and increased impedance in endometrial and subendometrial vasculature compared to healthy ovulatory women. Moreover, the pulsatility index of the main uterine arteries during the late follicular phase was reported to be elevated in women under clomiphene citrate treatment compared to women with natural cycles (Hsu et al., 1995). A study from Japan has shown that uterine perfusion in clomiphene citrate stimulated cycles is significantly lower than natural cycles on the day of ovulation (Nakai et al., 2002). Miwa et al. (2009) reported significantly higher resistive index in uterine radial arteries in women with thin endometrium than in women with normal thickness endometrium during different phases of the menstrual cycle. Takasaki and co-workers (2010) detected improvement in endometrial thickness, and uterine radial artery RI with the use of different vasodilator agents (vitamin E, L-arginine, and vaginal sildenafil acetate) in patients with thin endometrium (< 8 mm), and high radial artery RI. All this body of research evidence supports the notion that there is high uterine vascular resistance and impaired endometrial blood flow in women with clomiphene citrate-associated thin endometrium, and reversing this effect might help to improve endometrial thickness (Gutarra-Vilchez et al., 2014).

Vaginal administration of the sildenafil would achieve high concentration at the endometrium, meanwhile avoiding the well-known systemic side effects of sildenafil such as headache, hypotension, and flushing (Shanmugam et al., 2014). It is worth mentioning that only one of 36 patients who received sildenafil vaginal gel developed headache, with no other side effects reported.

Previous studies have used vaginal sildenafil in the form of suppositories that were prepared from oral tablets (Sher and Fisch 2002; Richter et al., 2007; Das et al., 2009; Takasaki et al., 2010; Malinova et al., 2013). However, there is no registered vaginal suppository form of sildenafil citrate in the market. In addition, vaginal suppositories have poor residence time (Brown et al., 1997), can be messy, and may not be able to deliver the exact medication dose (Bernkop-Schnürch and Hornof, 2003; Baloglu, et al., 2009). To avoid these disadvantages, we thought of applying sildenafil through a more retensive vaginal administration system (Merabet et al., 2005).

In the present study, we used an in-situ gelling drug delivery system for the vaginal administration of sildenafil. Our gel was based on two main polymers mixed together. Pluronic P-127 is a thermosensitive polymer, which changes from aqueous solution to viscous gel at body temperature (Arranja et al., 2014). Hydroxy Ethyl Cellulose is a mucoadhesive polymer increasing retention time of the gel in the vagina (Baloglu et al., 2009). These characters of our gel mixture allow more uniform distribution of sildenafil along vaginal absorptive surfaces, and release of the drug at more predictable rate for a longer time period (Baloglu et al., 2009). It is notable in our study that a significant increase in endometrial thickness, and uterine blood flow occurred with shorter total duration of sildenafil administration (only 5 to 6 days), in comparison to around 10 days in previous studies, which used the sildenafil vaginal suppositories formulation. In addition, we used less frequency dosing per day (two applications of 5 gm gel per day, each containing 50 mg of sildenafil), in comparison to 4 times per day in case of vaginal suppositories, each containing 25 mg/ day.

In conclusion, we have shown that sildenafil vaginal gel significantly increased endometrial thickness, uterine blood flow and may increase pregnancy rate in anovulatory patients with clomiphene citrate failure due to thin endometrium. The mucoadhesive vaginal gel formulation of sildenafil allowed shorter duration of drug application, and less frequent administration per day.
